# Identification of lesion subtypes in biopsies of ductal carcinoma in situ of the breast using biomarker ratio imaging microscopy

**DOI:** 10.1038/srep27039

**Published:** 2016-06-01

**Authors:** Andrea J. Clark, Howard R. Petty

**Affiliations:** 1 Department of Ophthalmology and Visual Sciences, 1000 Wall Street, University of Michigan Medical School, Ann Arbor, MI, USA

## Abstract

Although epidemiological studies propose aggressive and non-aggressive forms of ductal carcinoma *in situ* (DCIS), they cannot be identified with conventional histopathology. We now report a retrospective study of human biopsy samples using biomarker ratio imaging microscopy (BRIM). Using BRIM, micrographs of biomarkers whose expression correlates with breast cancer aggressiveness are divided by micrographs of biomarkers whose expression negatively correlates with aggressiveness to create computed micrographs reflecting aggressiveness. The biomarker pairs CD44/CD24, N-cadherin/E-cadherin, and CD74/CD59 stratified DCIS samples. BRIM identified subpopulations of DCIS lesions with ratiometric properties resembling either benign fibroadenoma or invasive carcinoma samples. Our work confirms the existence of distinct subpopulations of DCIS lesions, which will likely have utility in breast cancer research and clinical practice.

Ductal carcinoma *in situ* (DCIS) of the breast is the most common type of non-invasive breast cancer. In DCIS, epithelial cells proliferate within ducts, which are surrounded by a double layer of myoepithelial cells and basement membranes. Although it is believed that DCIS lesions become invasive breast cancer, this has not been proven, nor has the fraction of DCIS cases progressing to invasive cancer been established. The introduction of mammography led to a sharp increase in the number of DCIS cases. This increase, however, was not accompanied by a commensurate reduction in the number of advanced breast cancer patients. Several studies indicate that patients with insignificant disease are being treated^1^,^2^,^3^,^4^,^5^, which suggests the existence of both non-aggressive and aggressive forms of DCIS. Presently, it is not possible to stratify DCIS lesions according to aggressiveness with a precision sufficient to provide prognostic insight in patient care. To better classify DCIS lesions, we now introduce biomarker ratio imaging microscopy (BRIM). Ratio imaging microscopy has been used in calcium, membrane potential, intracellular pH, protein activation, fluorescence polarization, viscosity, proximity, and water permeability studies^6^,^7^,^8^,^9^,^10^. Two images are collected during ratio imaging microscopy: one increasing and one decreasing in intensity with the parameter of interest. Either one or two fluorescent labels may be used for ratioing^6^,^7^,^8^,^9^,^10^,^11^,^12^,^13^,^14^,^15^,^16^. During BRIM fluorescence images of two biomarkers are collected at distinct wavelengths wherein the expression of one biomarker increases with tumor aggressiveness while the second decreases with aggressiveness. By dividing the former by the latter, high contrast images linked with tumor aggressiveness are created. Moreover, optical artifacts due to variations in sample thickness disappear. Our work identifies DCIS lesions exhibiting high or low levels of ratiometric biomarker expression linked with tumor aggressiveness.

## Results

**Figure 1:**
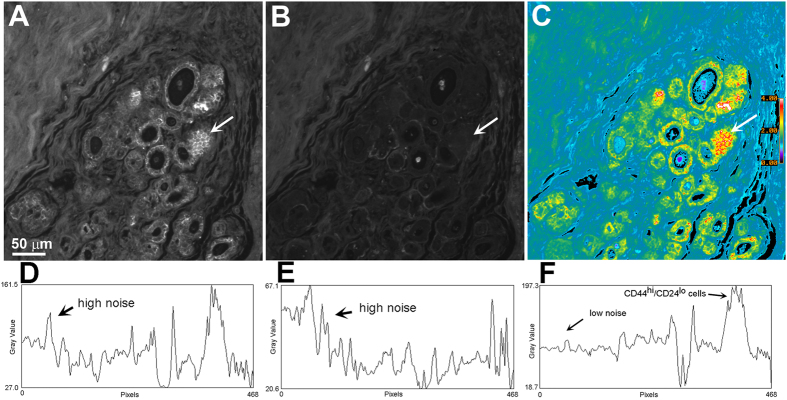
Illustration of BRIM. A DCIS section was labeled with anti-CD44 (**A**) and anti-CD24 (**B**). (Panels **A,B**) were prepared identically. (Panel **C**) reveals intraductal CD44^hi^/CD24^lo^ cells at high contrast. The white arrows identify a region of CD44^hi^/CD24^lo^ cells that are included in the quantitative line profile analyses of (panels **D–F**). (Panels **D–F**) show quantitative line profile analyses (the line profile extends from the right to left hand sides of the image at the level of the arrow). Noise reduction and contrast enhancement are seen in the ratio image of (panel **F**). The pseudocolor image in (panel **C**) is scaled as indicated by the bar on the right side. (Distance scale range is shown on the lower left side of (panel **A**).

To illustrate BRIM, we localized CD44^hi^/CD24^lo^ cells in DCIS pathology samples. CD44 and CD24 are cell surface adhesive proteins participating in proliferation and differentiation^17^. Importantly, CD44^hi^/CD24^lo^ cells have been reported to represent a population of breast cancer stem cells^18^, which were herein visualized by ratioing CD44 (numerator image) against CD24 (denominator image). Figure 1A–C shows: CD44, CD24, and CD44^hi^/CD24^lo^ images, respectively. The presence of high ratio cells in the ducts should be noted in Fig. 1C and Supplementary Fig. 1D. Quantitative line profile analyses of Fig. 1A–C are shown in panels D–F, respectively. These data illustrate the improvements provided by BRIM. For example, note that the parallel increases in CD44 and CD24 intensity seen in the region labeled “high noise” in Fig. 1D,E cancel out during ratioing, thus highlighting CD44^hi^/CD24^lo^ cells. However, CD44^hi^/CD24^lo^ cells could not be observed in a sub-population DCIS samples (see below).

**Figure 2:**
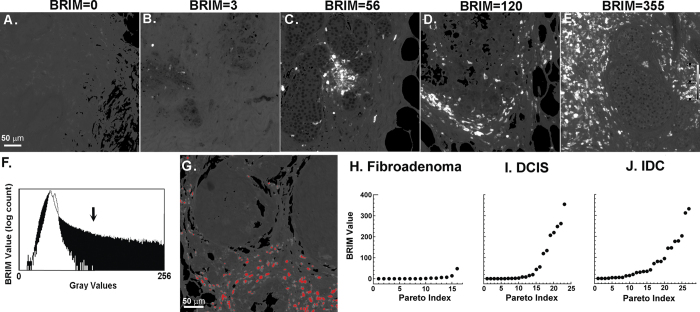
Stratification of DCIS samples using tissue sections stained with anti-CD74 and anti-CD59. A broad range of stromal cell ratio values were observed for samples from five DCIS patients (panels **A–E**). To quantify these data, further image processing was performed. The ratio of each pixel in a sample of normal tissue is shown in the white plot of (panel **F**) whereas an identical plot of DCIS tissue is shown as black in this panel. This diagram shows that gray values of >130 (see arrow) are only found in the DCIS sample. (Panel **G**) shows pixels with gray values of >130 (color-coded red), which confirms that stromal cells account for high BRIM values. The ascending Pareto plots of (panels **H–J**) show electronic counts of CD74^hi^/CD59^lo^ particles (ordinate) versus the data point index (abscissa). Each dot represents the BRIM score of one patient. Samples from simple fibroadenoma patients (**H**) display little or no signals (N = 16). DCIS samples (**I**) segregate into two populations: those scoring near the level of fibroadenomas and those samples scoring highly for the biomarker ratio pair (N = 23). High levels of statistical significance (P < 0.0001) are seen in comparing the low BRIM with high BRIM samples. The high BRIM scores can be seen for the DCIS population and IDC patient samples (**J**) (N = 26). Quantitative BRIM counts are shown at the top of (panels **A–E**). A ratio scale is given on the far right of (panel **E**) (20× objective).

On the basis of prior biomarker research^19^,^20^,^21^, we studied CD74^hi^/CD59^lo^ cells in DCIS samples. Overexpression of CD74, the HLA class II γ chain, and underexpression of CD59, a complement regulatory protein, are linked to poor patient outcomes^20^,^21^. Figure 2A–E shows the widely varying ratiometric intensities of five DCIS samples labeled for CD74 and CD59 biomarkers. Micrographs were next quantified for statistical purposes. We first compared pixel intensity histograms of control breast tissue (white region; low BRIM value) with DCIS tissue expressing aggressive biomarker properties (black region; high BRIM value); gray values >130 were only found in the DCIS sample (Fig. 2F). Figure 2G shows an image wherein gray values >130 were labeled red, which shows that stromal cells, not intraductal cells (Fig. 1C) were labeled. This threshold, based upon differences in clinical breast tissue samples, was applied in subsequent analyses. The results of this segmentation procedure were nearly indistinguishable from that obtained using a non-interactive Otsu algorithm (Supplementary Fig. 2). Figure 2I shows the number of high BRIM value particles per 20x micrograph for each DCIS patient. The inflection point found in the Pareto plot of panel I suggests two sample populations with mean particle counts of 4 ± 6 and 190 ± 100, which is further supported by Ashmann’s bimodality test^22^. These two populations were distinguishable at a high level of statistical significance (P < 0.0001). Quantitative data from simple fibroadenoma (5 ± 12) and IDC (76 ± 94) samples are shown as biological reference standards of benign and invasive breast neoplasms (Fig. 2H,J). Our studies demonstrate that BRIM stratifies DCIS samples. The IDC plot (Fig. 2J) was similar to DCIS samples (Fig. 2I). BRIM micrographs illustrating IDC staining are shown in Supplementary Fig. 3. The low BRIM DCIS subtype resembles the ratiometric biomarker properties of fibroadenoma samples whereas the high BRIM DCIS subtype more closely resembles IDC samples.

**Figure 3:**
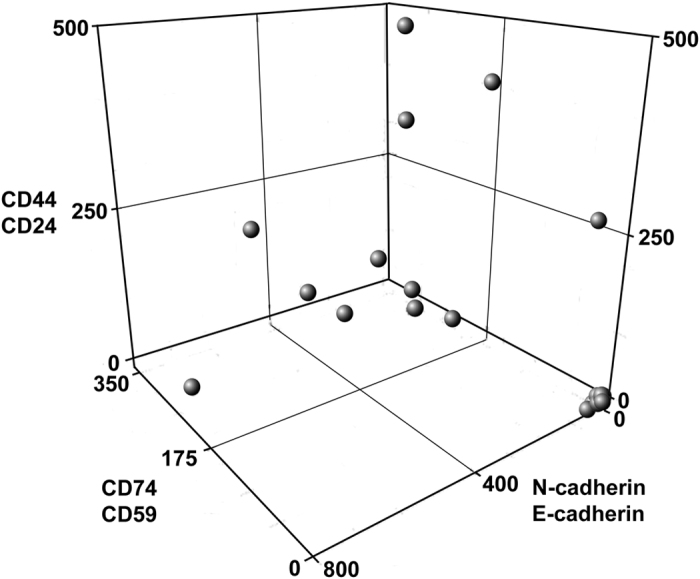
A three-dimensional rendering of all BRIM data for DCIS patients. Values of the parameters CD74/CD59 (z axis), CD44/CD24 (x axis) and N-cadherin/E-cadherin (y axis) are plotted for each DCIS patient. Some samples^18^ had BRIM values far from the origin (0, 0, 0). Of the samples near the origin, 5 had scores of <50 for CD74/CD59 and 0–2 for the other two parameters. Note that DCIS samples near the walls of the three-dimensional plot could be interpreted as false-negatives if only one BRIM parameter was measured.

As DCIS lesions may become aggressive independently of stromal cell phenotype, we examined additional biomarkers. As the endothelial-mesenchymal transition (EMT) is believed to participate in metastasis^23^, we quantified N-cadherin^hi^/E-cadherin^lo^ labeling. Similarly, CD44^hi^/CD24^lo^ cells also contribute to aggressiveness^18^. CD44^hi^/CD24^lo^ and N-cad^hi^/E-cad^lo^ cells were found to stratify DCIS tissues lesions (Supplementary Fig. 4)(for examples of relevant micrographs, see Fig. 1, Supplementary Figs 1 and 2). Figure 3 shows a three dimensional plot of CD74^hi^/CD59^lo^, CD44^hi^/CD24^lo^ and N-cad^hi^/E-cad^lo^ BRIM findings for each patient. This plot reveals a low BRIM DCIS sub-population near the origin (0, 0, 0) and a second subtype far from the origin. Although no significant correlation between BRIM subtype and nuclear grade or estrogen receptor status was observed, the average age of patients whose samples had low BRIM scores (50 yrs) was significantly (P < 0.05) less than those with high BRIM scores (64 yrs) (Supplementary Table 1), which is consistent with recent studies^24^. Within this dataset 5 samples (22%) had scores of 0–2 for CD74^hi^/CD59^lo^ and CD44/CD24 as well as <50 for N-cad^hi^/E-cad^lo^ (this higher score corrects for N-cad^hi^/E-cad^lo^ stromal cells), suggesting a low level of aggressive biomarker labeling.

To illustrate the advantages of BRIM over conventional fluorescence imaging, we compared BRIM results with matched conventional fluorescence micrographs of numerator images. Supplementary Fig. 5 shows quantitative DCIS results comparing particle counts of N-cadherin images with particle counts of matched N-cad^hi^/E-cad^lo^ images. Although some level of correlation is expected because N-cad images are incorporated in N-cad^hi^/E-cad^lo^ images, statistical analysis indicates that these two measures only weakly correlate (R = 0.61). BRIM findings of N-cad^hi^/E-cad^lo^ cells substantially differ from those of conventional fluorescence microscopy (N-cad^hi^ cells). Similarly, weak correlations were observed for comparisons of CD44 versus CD44^hi^/CD24^lo^ (R = 0.76) and CD74 versus CD74^hi^/CD59^lo^ (R = 0.76). We speculate that analysis of a single biomarker may be insufficient to provide reliable prognostic information, and that multi-dimensional BRIM may be useful in breast cancer research and patient management.

## Discussion

Although screening tools for cervical and colorectal cancer reduced the incidence of advanced forms of these diseases, screening mammography has not yielded a similar reduction in advanced breast cancer^1^,^2^,^3^. Esserman and colleagues^2^,^5^ have proposed that DCIS subtypes differing in aggressiveness exist. Our BRIM studies using biomarkers of breast cancer aggressiveness have identified DCIS subtypes thereby confirming the postulate of Esserman and others regarding the heterogeneity of DCIS subtypes. Our study also provides the first robust images of CD44^hi^/CD24^lo^ cells within human tissue samples. The detection of these cells should accelerate the search for new anti-stem cell cancer drugs. As CD44^hi^/CD24^lo^ cells may contribute to the failure of chemotherapy and radiotherapy^18^,^25^, their abundance and/or ratiometric values may provide predictive information. The identification of intraductal N-cad^hi^/E-cad^lo^ cells indicates that the EMT accompanies human cancer, not just murine cancer^26^. In some DCIS cases a sub-population of CD74^hi^/CD59^lo^ stromal cells can be observed. We speculate that stromal cells may participate in disease, as others have suggested^27^,^28^.

As a microscope-based tool, BRIM recognizes tumor cell heterogeneity, and permits biomarker detection at comparatively high concentrations within organelles. For image ratioing, the signal intensity must be linear with respect to biomarker number. Hence, enzyme-linked amplification methods are inappropriate due to their non-linear properties^29^. This difficulty is avoided by fluorescence microscopy. Variations in cell shape, size, and section thickness influence a sample’s perceived brightness. These optical path-length artifacts are removed by ratio imaging microscopy^6^,^7^. As instrument-dependent factors are present in the numerator and denominator, they are also removed by image ratioing, thereby improving standardization. Finally, noise due to sample loss during processing, knife chatter during sectioning and others are removed by ratioing (e.g., the upper left corner of Fig. 1A). Hence, BRIM overcomes many drawbacks of conventional histopathology.

As our samples originated from mastectomies without patient follow-up, we cannot know with certainty the lesions that would have progressed to life-threatening cancer. However, the association between the low BRIM scores of DCIS and fibroadenoma compared to the high BRIM scores of DCIS and IDC support the clinical relevance of these DCIS subtypes. The ability to stratify DCIS lesions and to identify potentially non-aggressive and aggressive lesions raises important issues in addressing overtreatment in breast cancer. BRIM is particularly attractive because it could be integrated into clinical pathology practices. Moreover, this approach may be useful in the cytologic study of aspirates in breast cancer and in peritoneal fluids in ovarian cancer. As overdiagnosis has also been reported for prostate, lung and other cancers^30^, BRIM may be broadly useful in cancer research and provide practitioners with important prognostic information in patient management.

## Methods

### Study Design and Patient Samples

In this study we used BRIM to evaluate the co-expression of biomarkers correlating and anti-correlating with breast cancer aggressiveness in a retrospective study of DCIS samples. We used fibroadenoma of the breast and invasive ductal carcinoma (IDC) to compare with DCIS samples. Formal-fixed paraffin-embedded (FFPE) pathology samples were purchased from the National Disease Research Interchange (NDRI), a National Resource Center (Bethesda, MD) and the Cooperative Human Tissue Network (CFTN) (Columbus, OH; Philadelphia, PA; and Nashville, TN). Samples were from mastectomies of females aged 37–96 years after informed consent was obtained from all subjects. As the tissues examined in this study were not needed for patient care, it is possible that small DCIS lesions were not adequately sampled. Samples from NDRI were collected between Nov. 2004 and Dec. 2014. The use of human material was in accordance with the Declaration of Helsinki on the use of material in scientific research. These studies were approved by the University of Michigan’s IRB.

### Biomarkers

Biomarkers were selected based upon their relative changes in expression in normal tissue vs. IDC. Although a high dynamic range of intensities is desirable, intensity differences causing division by zero errors are unhelpful. Punctate biomarker images (e.g., ribosomal biomarkers) and biomarkers that translocate between organelles (e.g., gene regulatory proteins) are also unsuitable for ratioing. The biomarkers used in this study have been previously reported, as described above. Our studies focus on the biomarker pairs: CD74^hi^/CD59^lo^, CD44^hi^/CD24^lo^ and N-cad^hi^/E-cad^lo^, which are plasma membrane proteins.

As antibodies may differ in their titer, epitope recognition, and binding after antigen retrieval, it is important to use matched antibodies in BRIM. The primary antibodies used in these studies were: Ms anti-N-Cadherin (Abcam ab98952), Rb anti-E-Cadherin (Abcam ab15148), Ms anti-CD74 (Abcam ab9514), Rb anti-CD59 (Abcam ab133707), Rb anti-CD44 (Abcam ab41478), Ms anti-CD24 (Biolegend 311102). The secondary antibodies used in this study were: Gt anti-Ms Alexa 488 (Invitrogen A11029) and Dk anti-Rb Alexa 568 (Invitrogen A10042). These antibodies were typically used at a 1/100 dilution.

### Histochemistry of tissue sections

Sections of paraffin-embedded samples were cut into 5 μm sections. Sections were stained with hematoxylin/eosin (H&E). For immunofluorescence staining, sections were deparaffinized and re-hydrated by sequential incubation in a graded ethanol series. After rehydration in PBS, sections were subjected to heat-mediated antigen retrieval in 10 mM citric acid buffer, pH 6.0. Sections were blocked using 10% non-fat dried milk for 1.5 hr. at room temperature. After blocking procedures, sections were incubated with 2 μg/mL of antibody diluted in 1% BSA in PBS overnight at 4 °C. After incubation, the sections were washed with PBS. Finally, the sections were incubated with fluorescently labeled secondary antibody for 1.5 hr., washed with PBS, and then mounted in 90% glycerol in PBS. The H&E and immunofluorescence images were acquired from serial sections from each block.

### Optical microscopy

Fluorescence microscopy was performed using a Nikon TE2000-U inverted microscope (Nikon, Melville, NY) and an Andor iXon camera (Andor Technology, Belfast, Northern Ireland) with a 100 W mercury lamp. Ratio imaging experiments were performed as we have described^13^,^14^. To avoid cross-talk between the emission wavelengths of the fluorescent tags, band-pass filters with the “sharpest” cut-offs and greatest out-of-band reflectances were used (Chroma ET-fluorescein filter set (#49011) and Chroma ET-Cy3/rhodamine filter set (#49004) (Chroma Tech. Corp., Bella Falls, VT)). These zero pixel shift optics were chosen for their high performance in ratio imaging. For most experiments, 20 images, each acquired for 0.2 sec., were averaged. The electron multiplying charge coupled device chip was cooled to −85 °C. Typical camera settings were: multiplication gain, 150; vertical shift speed, 3.04 msec./pixel and 14-bit digitization at 10 MHz. Images were captured with Metamorph and processed with MetaFluor software (Molecular Devices, Downingtown, PA) to calculate image ratios. Micrographs were evaluated using ImageJ software. Segmentation was performed with the ISODATA (iterative self-organizing data analysis) technique. A local implementation of the Otsu algorithm^31^ was used for comparison. (Global segmentation is recommended over local implementations of thresholding approaches, as fat droplets in mammary tissue may be misinterpreted by software.) Ratiometric images at a gray value of >130 were quantified by counting the number particles five or more pixels in size. The highest BRIM value from each patient’s micrographs was plotted. Several micrographs from each patient sample were analyzed because some micrographs contained lower numbers of cells, for example, due to an abundance of fatty tissue. Data were displayed using two-dimensional (KaleidaGraph, Synergy Software, Reading, PA) or three-dimensional (Plotly, Montreal, Quebec, CA) graphing software.

### Statistics

*In vitro* data are presented as the mean ± sd to describe the dispersion of the data. Data were evaluated with Student’s t-test. Welch’s t-test, which is less susceptible to departures from a normal distribution, yielded indistinguishable results.

## Additional Information

**How to cite this article**: Clark, A. J. and Petty, H. R. Identification of lesion subtypes in biopsies of ductal carcinoma in situ of the breast using biomarker ratio imaging microscopy. *Sci. Rep.* **6**, 27039; doi: 10.1038/srep27039 (2016).

## Supplementary Material

Supplementary Information
