# Supplementary material for: Identification of lesion subtypes in biopsies of ductal carcinoma in situ of the breast using biomarker ratio imaging microscopy
Source: Sci Rep. 2016 Jun 1;6:27039. doi: 10.1038/srep27039 (PMC4887986; doi:10.1038/srep27039)
Supplement: Supplementary Information [file srep27039-s1.pdf]

Supplementary Information

Identification of lesion subtypes in biopsies of ductal carcinoma in situ of the breast using biomarker ratio imaging microscopy

Andrea J. Clark and Howard R. Petty

Contents

Supplementary Figure 1

Supplementary Figure 2

Supplementary Figure 3

Supplementary Figure 4

Supplementary Figure 5

Supplementary Table 1

Supplementary Figure 1

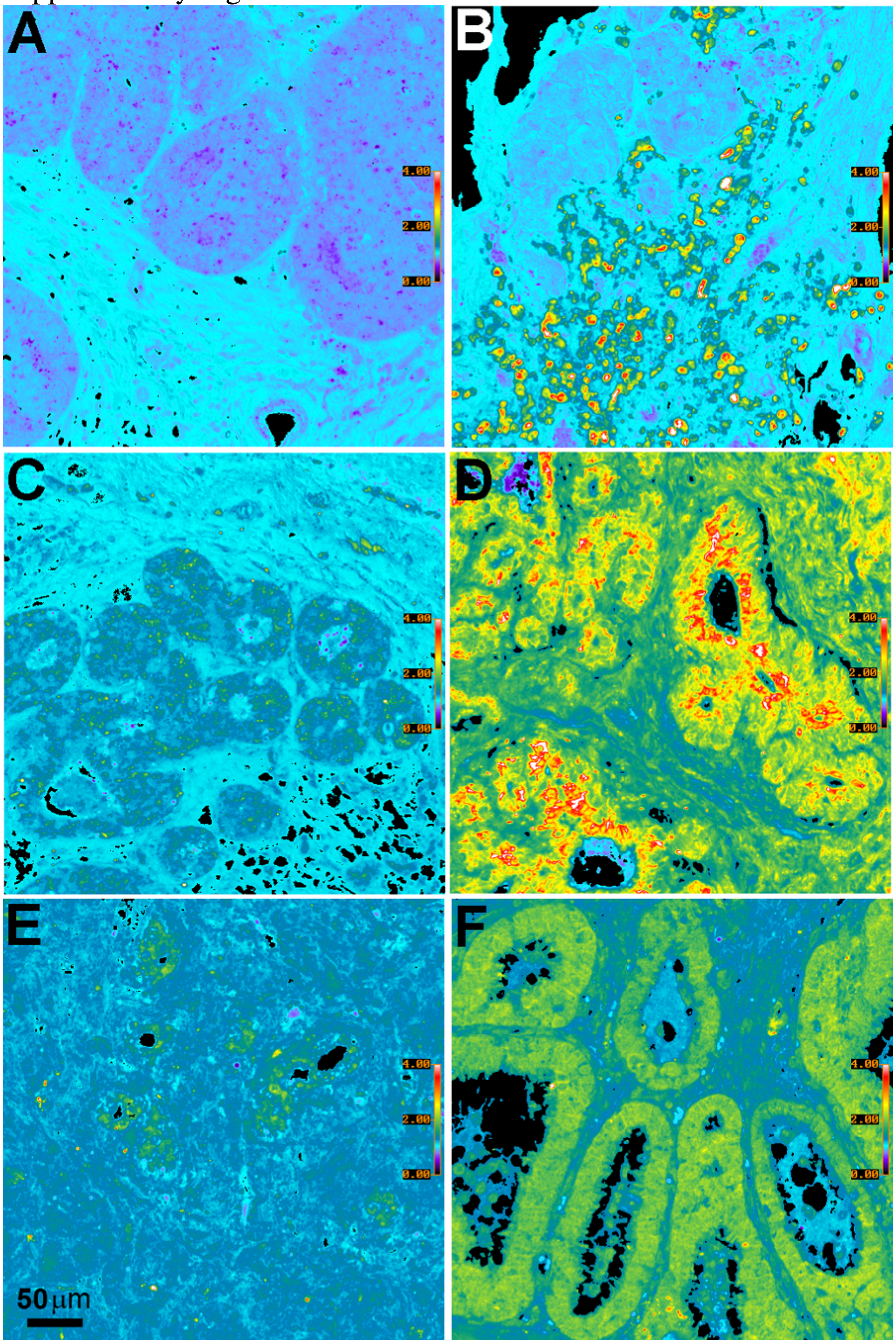

Supplementary Figure 1. Examples of pseudocolor ratio images of DCIS tissue samples are shown. This figure illustrates DCIS tissue samples with low (A, C, E) and high (B, D, and F) image ratios for the biomarkers: CD74/CD59 (A, B), CD44/CD24 (C, D), and N-cadherin/E-cadherin (E, F). It should be noted that stromal and epithelial cells are positive in panels B and D, respectively. (Ratio bars are given along the right hand side of each figure.)

Supplementary Figure 2

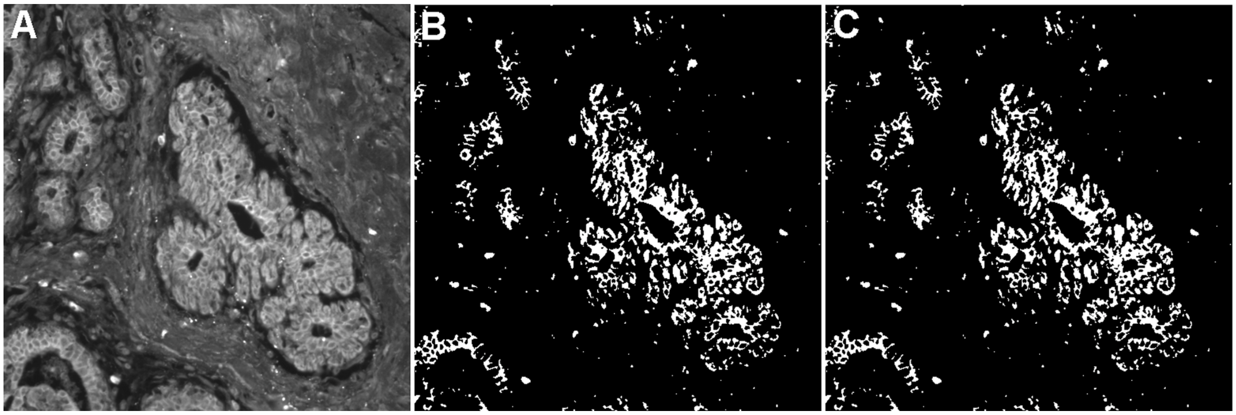

Supplementary Figure 2. Examples of image segmentation producures are shown. A ratiometric N-cadherin/E-cadherin image of a DCIS sample is shown (panel A). In panel B, the ratio image of panel A was segmented using the ISODATA algorithm. Panel C shows the same image segmented using the Otsu algorithm. The threshold was interactively selected by comparison with normal and simple fibroadenoma samples in panel B whereas in threshold was automatically optimized in panel C. Very similar results were obtained.

Supplementary Figure 3

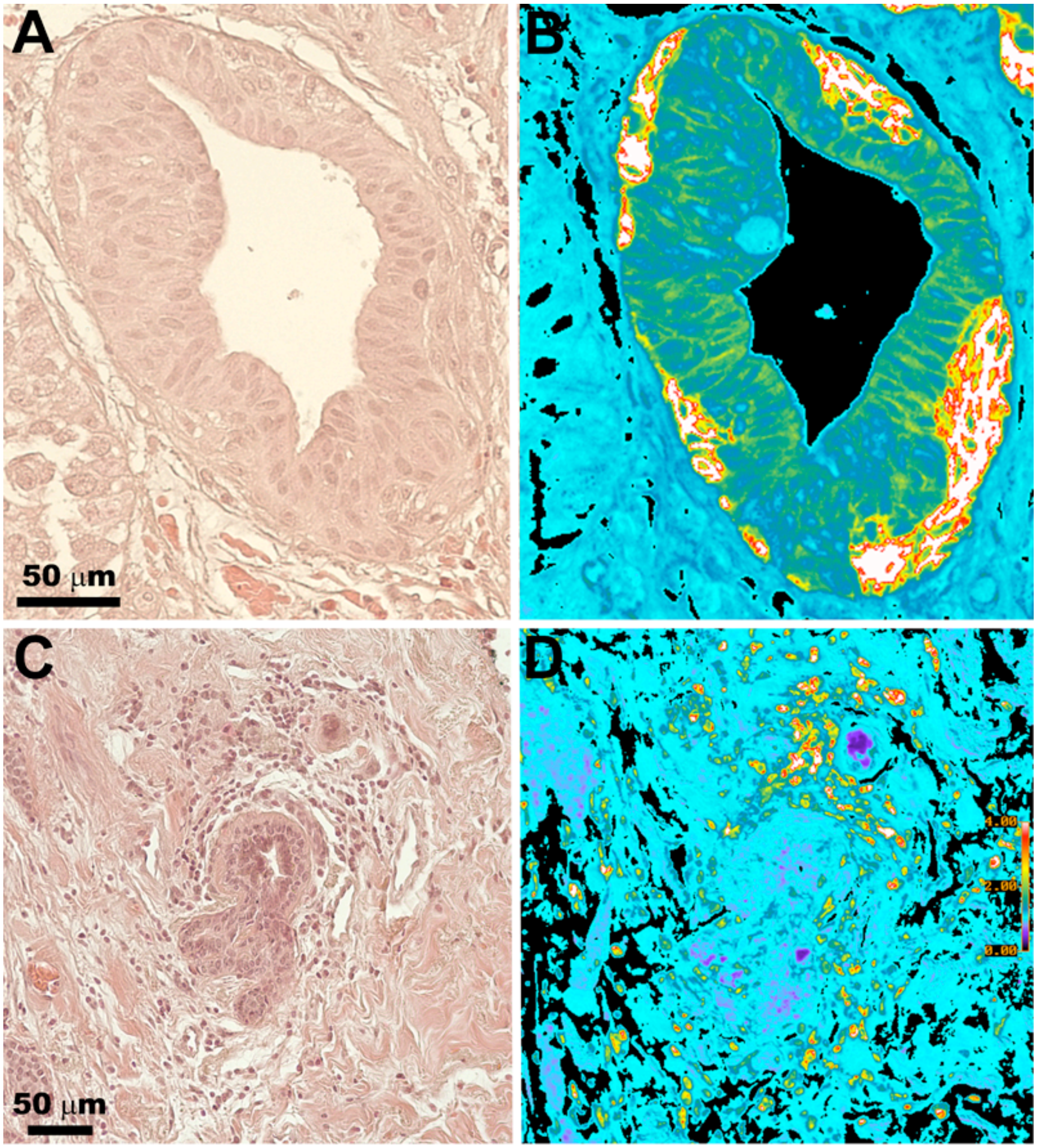

Supplementary Figure 3. High BRIM ratios are found for IDCs. H&E (A, C) and BRIM (B, D) micrographs of tissue sections of an IDC. In each pair of micrographs, images of the same tissue region for two nearby tissue sections are shown. An H&E stain of an IDC tissue sample is illustrated in panel A. Panel B shows the same region stained for CD44 and CD24 after BRIM processing. Note that the highest BRIM ratio cells at the perimeter of the duct do not have a columnar morphology. Panels C shows an H&E stain of an IDC sample. Panel D shows a serial section of the same patient sample stained with CD74 and CD59 then processed. $CD74^{hi}/CD59^{lo}$ cells are apparent. (A, C; bar = 50 μm)

Supplementary Figure 4

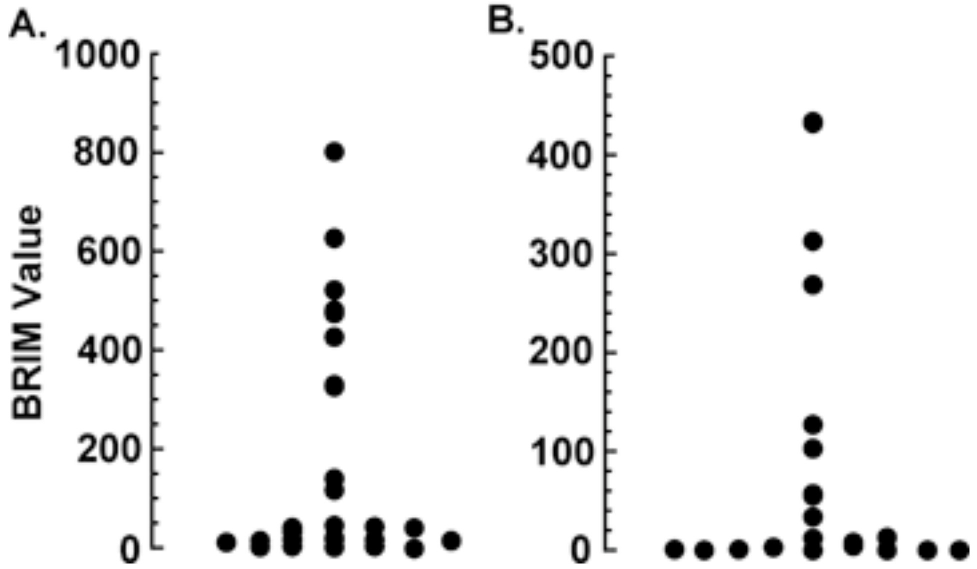

Supplementary Figure 4. Stratification of DCIS samples using N-cadherin/E-cadherin (A) and CD44/CD24 (B) ratiometric images are shown. Each dot represents the BRIM value of one patient. A group of patients had very low BRIM values, thus accounting for the extensive number of cases near the baseline. (Some data points may overlap in these scattergrams.)

Supplementary Figure 5

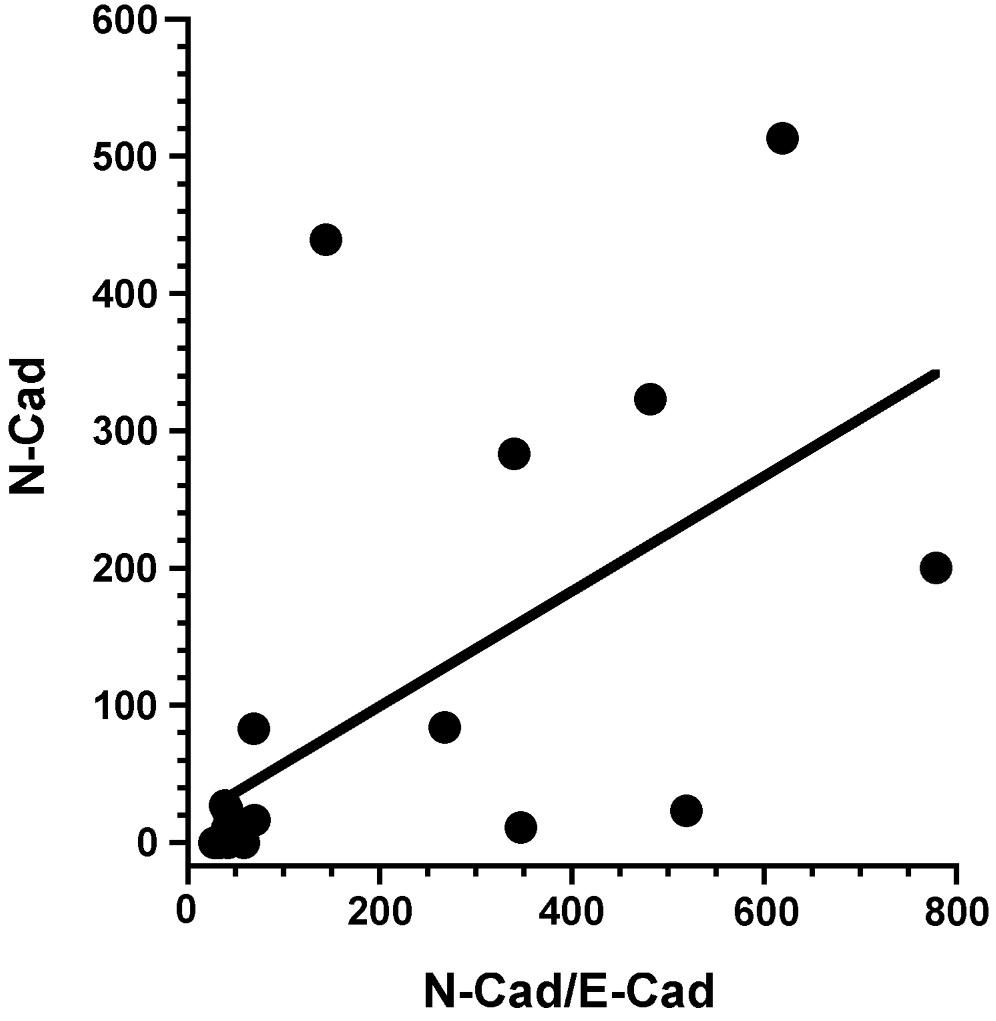

Supplementary Figure 5. Comparison of N-cadherin/E-cadherin ratiometric results (abscissa) with single channel assessments of N-cadherin (ordinate) of DCIS samples. The N-cad and N-cad/E-cad results are from matched sets. The correlation coefficient (R = 0.61) indicates that these two measures weakly correlate. This weak correlation was largely due to samples poorly staining for N-cadherin. This plot also reveals that some cells with low N-cadherin levels nevertheless expressed high N-cadherin/E-cadherin ratios. (Data from two patients were excluded because their N-cad staining was too high to reliably measure.)

Supplementary Table 1. DCIS Patient Details

number	age	CD74/CD59	N-cad/E-cad[1]	CD44/24	architecture	grade	Antigen status[2]	BRIM status
1	50	263	117	486	comedo, cribriform, micropapillary	high	na	high
2	53	56	474	194	cribriform	intermediate	na	high
3	67	220	627	248	cribriform, solid	intermediate	na	high
4	44	3	481	211	solid	Low to intermediate	na	high
5	48	1	17	8	solid, cribriform, comedo	high	ER+, PR+	high
6	54	207	802	55	comedo	high	ER-	high
7	46	120	140	432	solid, micropapillary, mucinous	low	na	high
8	82	249	330	57	comedo, cribriform, micropapillary	high	ER+, PR+	high
9	52	0	31	9	solid, cribriform	high	ER+, PR-	high
10	64	44	522	434	cribriform, papillary	intermediate	ER+, PR+	high
11	59	134	326	313	comedo	high	ER-	high
12	56	355	41	55	solid, comedo	intermediate to high	na	high
13	55	1	11	4	solid, cribriform	low	na	high
14	79	8	13	13	comedo	high	na	high

15	42	0	0	12	cribriform, papillary, solid, focal apocrine features	intermediate	na	high
16	38	15	15	34	Comedo, cribriform apocrine features	high	na	high
17	37	19	42	127	micropapillary	low	na	high
18	46	8	20	5	comedo, cribriform, solid	high	na	high
19	52	0	1	1	cribriform solid	intermediate	na	low
20	50	0	1	0	solid, cribriform	high	na	low
21	53	0	0	1	cribriform, micropapillary	intermediate	na	low
22	51	0	14	0	cribriform, solid	high	na	low
23	43	2	13	0	solid, cribriform	high	na	low

1) N-cad^{hi}/E-cad^{lo} cells may be both stromal cells and tumor cells. Thus, some background level of positivity is expected.

Intraductal N-cad^{hi}/E-cad^{lo} cells and BRIM values >50 indicate an aggressive phenotype. 2) na = not available.
